# TRAIL/DR5 pathway promotes AKT phosphorylation, skeletal muscle differentiation, and glucose uptake

**DOI:** 10.1038/s41419-021-04383-3

**Published:** 2021-11-16

**Authors:** Barbara Toffoli, Federica Tonon, Veronica Tisato, Giorgio Zauli, Paola Secchiero, Bruno Fabris, Stella Bernardi

**Affiliations:** 1 Institute for Maternal and Child Health, IRCCS “Burlo Garofolo”, via dell’Istria 65/1, 34137 Trieste, Italy; 2 Department of Medical, Surgical and Health Sciences, University of Trieste, Cattinara Teaching Hospital, Strada di Fiume 447, 34149 Trieste, Italy; 3 Department of Translational Medicine, University of Ferrara, Via Fossato di Mortara 70, 44121 Ferrara, Italy; 4 Dipartimento di Scienze dell’Ambiente e della Prevenzione, University of Ferrara, Via Corso Ercole I d‘Este 32, 44121 Ferrara, Italy

**Keywords:** Insulin signalling, Mechanisms of disease

## Abstract

TNF-related apoptosis-inducing ligand (TRAIL) is a protein that induces apoptosis in cancer cells but not in normal ones, where its effects remain to be fully understood. Previous studies have shown that in high-fat diet (HFD)-fed mice, TRAIL treatment reduced body weight gain, insulin resistance, and inflammation. TRAIL was also able to increase skeletal muscle free fatty acid oxidation. The aim of the present work was to evaluate TRAIL actions on skeletal muscle. Our in vitro data on C2C12 cells showed that TRAIL treatment significantly increased myogenin and MyHC and other hallmarks of myogenic differentiation, which were reduced by *Dr5* (TRAIL receptor) silencing. In addition, TRAIL treatment significantly increased AKT phosphorylation, which was reduced by *Dr5* silencing, as well as glucose uptake (alone and in combination with insulin). Our in vivo data showed that TRAIL increased myofiber size in HFD-fed mice as well as in db/db mice. This was associated with increased myogenin and PCG1α expression. In conclusion, TRAIL/DR5 pathway promotes AKT phosphorylation, skeletal muscle differentiation, and glucose uptake. These data shed light onto a pathway that might hold therapeutic potential not only for the metabolic disturbances but also for the muscle mass loss that are associated with diabetes.

## Introduction

Tumor necrosis factor (TNF)-related apoptosis inducing ligand (TRAIL) is a member of the TNF family of structurally related cytokines, which influence a variety of cellular functions including survival and death [1–3]. TRAIL is expressed in most of the cells and its best-known biological effect is the induction of apoptosis in different cancer cell lines while sparing the normal ones [4].

Over the past decade, TRAIL signaling has been linked to the development of obesity and diabetes [5]. The first studies establishing a role for TRAIL in diabetes were carried out in animal models of type 1 diabetes mellitus (T1DM), where TRAIL blockade significantly accelerated diabetes development and the degree of autoimmune inflammation [6]. Subsequent studies demonstrated that TRAIL inhibited the proliferation of diabetogenic T-cells [7], and that the development and progression of T1DM were reduced by adenovirus-mediated TRAIL delivery into pancreatic islets [8], or by treatment with recombinant TRAIL [9]. These results were ascribed to TRAIL contribution to the negative selection of autoreactive thymocytes—which could account also for its anticancer activity—[10], and suggested that TRAIL could be an important immune regulator, opposing T1DM development.

Further studies have extended TRAIL actions to T2DM development and progression. In this setting, TRAIL deficiency significantly increased body weight gain and impaired glucose tolerance [11]. Consistent with this, TRAIL treatment significantly improved insulin resistance in high-fat diet (HFD)-fed mice [12, 13]. This was associated not only with a reduction of low-grade inflammation but also with a significant reduction of body weight gain, total adiposity, and liver fat content [12–14]. Subsequent in vitro studies, aiming at providing mechanistic insights, showed that TRAIL inhibits glucose uptake and lipogenesis by downregulating key adipogenic transcription factors in adipocytes [15, 16], and that it significantly reduces lipid droplet accumulation in hepatocytes [13].

Although skeletal muscle plays a central role in the control of glucose metabolism, TRAIL direct actions on myoblasts/myocytes have not been characterized yet. Nevertheless, a few years ago we found that treatment with recombinant TRAIL significantly increased ex vivo palmitate oxidation in the skeletal muscle [12], suggesting a protective effect of TRAIL against mitochondrial dysfunction, lipid accumulation, and insulin resistance. Based on this background, the aim of the present study was to evaluate TRAIL effects on skeletal muscle.

## Materials and methods

### In vitro studies

Mouse C2C12 myoblasts (ECACC General Cell Collection, Merck, Darmstadt, Germany) were cultured as previously described [17]. D is for day of differentiation. Cells were used for experiments on the 3^rd^ day of differentiation (D3) or every day during this process (D0–D4). In the studies carried out between D0–D4, TRAIL treatment (10; 100 ng/mL) was renewed every 24 h.

### In vivo studies

Mouse skeletal muscles (quadriceps) were taken from two different animal study protocols [13, 18]. In the first study protocol, 8-week-old C57Bl6 male mice were fed with a HFD (60% of energy coming from fat) for 12 weeks. After 4 weeks of HFD, they were randomly allocated to saline (HFD group, *N* = 10) or to TRAIL (HFD + TRAIL group, *N* = 10) that was administered at the dose of 10 µg/mouse/week by intraperitoneal injection for 8 weeks [13]. An additional group of 8-week old C57Bl6 male mice was fed with a standard diet for 12 weeks and was the control group (CNT group, *N* = 10). In the second study protocol, 8-week-old db/db male mice (BKS.CG-M + / + LEPRDB/L; purchased from Charles Rivers Laboratories) were randomly allocated to saline (db/db group, *N* = 10) or TRAIL (db/db + TRAIL group, *N* = 10) that was administered at the dose of 15 ug/mouse by intraperitoneal injection twice per week per 12 weeks [18]. An additional group of 8-week-old BKS.CG-M DB/ + male mice was randomly allocated to saline (db/H group, *N* = 10) or TRAIL (db/H + TRAIL group, *N* = 5). All the details of the respective experimental protocols can be found in [13, 18]. At the end of the studies, animals were anesthetized by an IP injection of tiletamine/zolazepam (80 mg/kg) and the blood was collected from the left ventricle. Quadriceps were collected and either snap frozen or fixed in formalin for further analyses. In both studies, animals were kept at the Animal House of the Cluster in Biomedicine (C.B.M. S.c.r.l. Area science Park, Trieste, Italy). All animal procedures were approved by the Institutional Animal Care and Use Committee of the C.B.M. and by the Italian Ministry of Health (DM 17/2001 # A dd 02/02/2011 and # 712/2016-PR).

### Recombinant human TRAIL

Recombinant histidine-6-tagged human TRAIL (114-281) was produced in transforming bacteria BL21 with a pTrc-His6 TRAIL vector, as previously described [19]. Human TRAIL shares approximately 65% amino acid sequence homology with mouse TRAIL. It is active on mouse cells, where it binds to DR5 [1, 2].

### Dr5 silencing

The siRNA sequences directed to 2 different regions of the *Dr5* mRNA were purchased from Thermo Fisher Scientific (Waltham, MA, USA) (Silencer^®^ Select, Tnsrsf10b, ID s75264 and s75265). C2C12 myoblasts were seeded in 6- or 12-well plates and transfected with 100 nM of *Dr5* or control siRNAs (CTR, Silencer Select Negative Control #1) using RNAiMAX reagent and Opti-MEM Medium (Thermo Fisher Scientific) for 5 h. Subsequently, cells were allowed to grow in DMEM medium supplemented with 10% FBS and switched to differentiation medium after 48–72 h. Morphologic changes were evaluated under a light microscope at selected time points before mRNA isolation or protein extraction.

### Free fatty acids experiments

C2C12 cells were cultured for 24 h in a medium supplemented with BSA alone (control), or BSA-conjugated oleate (500 μM) or palmitate (300–500 μM) (Merck), ± TRAIL (1; 10; 100 ng/mL). Cell viability was assessed with the MTT assay (Alfa Aesar; #L11939; Thermo Fisher Scientific). Total intracellular lipid content was evaluated and visualized by Oil Red O staining and LipidTOX staining (Thermo Fisher Scientific).

### Glucose uptake studies

Once C2C12 cells differentiated on 24-well plates (D3), they were treated ± TRAIL (10; 100 ng/mL) for 24 h. Then, after a medium change, the cells were serum starved overnight and then glucose starved for 40 min. Subsequently, they were stimulated with or without insulin (1μM) for 20 min and then 2-Deoxyglucose (2-DG) (final concentration 1 mM) was added for 20 min. Glucose uptake was measured by using the Glucose Uptake Colorimetric Assay kit (Merck, MAK083) following manufacturer’s instructions.

### Gene expression quantification by real-time RT-PCR

RNA was extracted from skeletal muscles (100 mg) or C2C12 cells. Details of RNA extraction, treatment with DNase, and cDNA synthesis can be found in ref. [18]. The expression of genes related to metabolism (*Cpt1b*, *Glut4, Pepck1, Pgc1α, Pgk1, Pparg*), differentiation (*Myod*, *Myog, Myh4*), and atrophy (*Mafbx* and *Murf1*) was analyzed by RT-qPCR using the SYBR Green system (Thermo Fisher Scientific). *Dr5* (*Tnfrsf10b*) expression was evaluated with the TaqMan Gene Expression Assay. Fluorescence for each cycle was quantitatively analyzed by StepOnePlus real-time PCR system (Applied Biosystems, Thermo Fisher Scientific). Gene expression was normalized to *Gapdh*, *Rpl27* or *18s*, and reported as a ratio compared with the level of expression in untreated controls, which were given an arbitrary value of 1. Primer sequences used with the SYBR Green system are reported in Supplementary Table 1.

### Stainings

To measure MyHC immunofluorescence, C2C12 cells were grown on collagen pre-coated glass coverslips. After fixation and permeabilization, they were incubated with a mouse anti-Myosin Heavy Chain antibody (1:100; #05-716, Merck) overnight at 4 °C, and then with a fluorescent goat anti-mouse antibody (1:400; Alexa Fluor 488, Thermo Fisher Scientific) for 45 min at RT. After DAPI staining, coverslips were mounted with the Prolong Gold Antifade Mountant (Thermo Fisher Scientific) and the images were captured on a Leica DM-2000 microscope (Leica Microsystems, Wetzlar, Germany). Total cell nuclei and nuclei within myotubes were counted using the NIH ImageJ software. The fusion index corresponds to the ratio between the number of nuclei in a MyHC-positive myotube (with > 3 nuclei) and the total number of nuclei. To assess *Dr5* silencing effect, cells were incubated with a rabbit anti DR5 antibody (1:100; #ab8416, Abcam, Cambridge, United Kingdom), in addition to the anti-MyHC antibody (1:200), overnight at 4 °C. Mitochondrial superoxide levels in C2C12 cells were visualized by MitoSOX Red Staining (Thermo Fisher Scientific) following manufacturer’s instructions. In order to evaluate myofiber cross sectional area, 4-µm muscle sections were stained with hematoxylin and eosin (H/E). Fiber size was determined for at least seven captured images from each mouse. Areas were traced manually and computed by ImageJ software. All the measurements were performed blindly.

### Western blot analysis

Proteins were extracted from whole-cell lysates or skeletal muscles with Ripa lysis Buffer (Merck). A total of 20–40 μg of protein were separated on SDS/PAGE and transferred onto nitrocellulose membranes by semidry transfer. Then, they were blocked and incubated overnight at 4 °C with primary antibodies (Supplementary Table 2). The next day, membranes were incubated with secondary antibodies HRP and immunoreactivity was detected using an enhanced chemiluminescence kit (Bio-Rad, Hercules, CA, United States) and exposure to Chemidoc Imaging System (Bio-Rad).

### Statistical analysis

Statistical analysis was performed with GraphPad Prism 8. The Shapiro–Wilk test was used to check the distribution normality of continuous variables. Continuous variables are reported as median with IQ range. Continuous variables were compared using ANOVA and Tukey post-hoc test (or Kruskal–Wallis with Dunn’s post-hoc test, based on data distribution). In case of two groups, we used the *t*-test (or the Mann–Whitney).

## Results

### TRAIL does not reduce lipid accumulation on skeletal myotubes

**Fig. 1:**
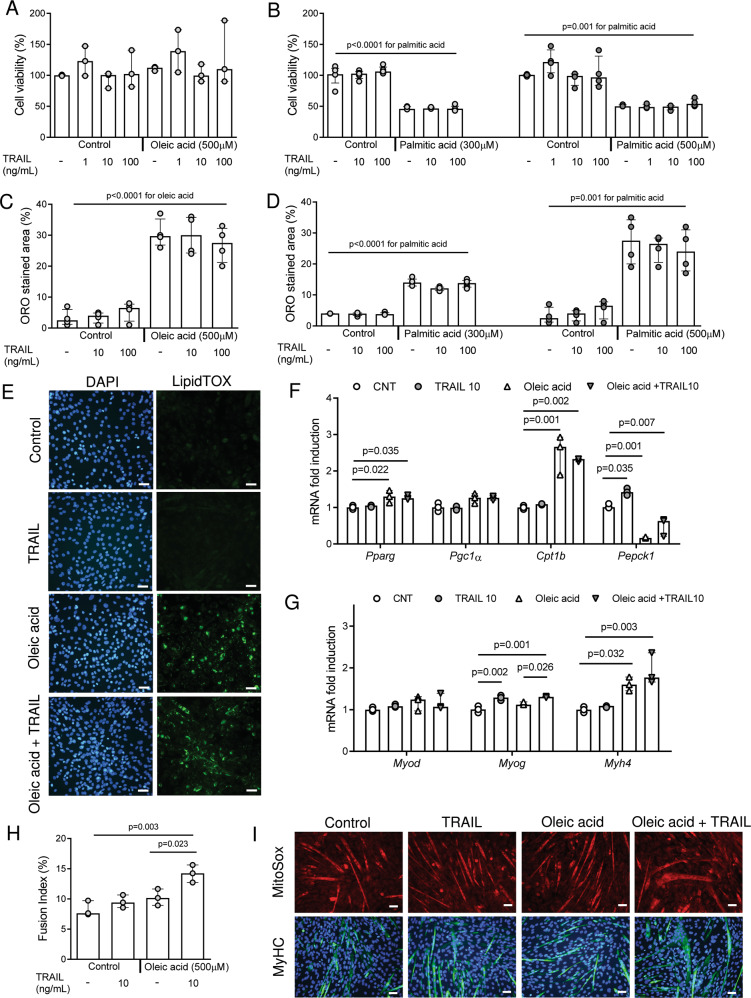
TRAIL does not modify lipid accumulation but stimulates myogenic markers. Experiments were conducted in C2C12 cells on D3 (3^rd^ day of differentiation), after 24 h exposure to oleic acid or palmitic acid ± TRAIL. **A**, **B** Cell viability. **C**, **D** Quantification of lipid droplet content (expressed as percentage of Oil red O stained area). **E** Representative images of C2C12 cells stained with DAPI (left panels) and LipidTOX (right panels) in presence or absence of oleic acid (500 µM) ± TRAIL (100 ng/mL), scale bar 50 μm. **F**, **G** Gene expression of *Pparg*, *Pcg1α*, *Cpt1b*, *Pepck1, Myod*, *Myog*, and *Myh4*. Gene expression is reported as mRNA fold induction normalized to the mRNA level of CNT. **H** Fusion index quantification. The fusion index corresponds to the ratio between the number of nuclei in a MyHC-positive myotube (with > 3 nuclei) and the total number of nuclei. **I** Representative images of C2C12 cells stained with MitoSox (red staining, top panels) and anti-MyHC (green staining, bottom panels) in the presence or absence of oleic acid (500 µM) ± TRAIL (10 ng/mL), scale bar 50 μm. Results were obtained by 3–5 independent experiments and are presented as median with interquartile range. Significance was assessed with ANOVA and Tukey.

Previous works showed that TRAIL treatment reduced lipid accumulation [13]. Our first experiments aimed to determine whether TRAIL reduced it also on skeletal muscle. To this end, C2C12 cells were cultured with either palmitate or oleate, in the presence or absence of TRAIL, on D3 (the 3^rd^ day of differentiation). Oleic acid and TRAIL had no effect on C2C12 cell viability, while palmitate significantly impaired it (Fig. 1A, B). TRAIL did not reduce lipid droplet accumulation, as assessed by Oil Red O and LipidTOX staining (Fig. 1C–E). In addition, TRAIL had no effect on the expression of *Pparg*, *Pgc1α*, and *Cpt1b*, which were upregulated by oleate, while it significantly increased *Myog* expression with and without oleate (Fig. 1F–G). Likewise, the C2C12 cells that were co-treated with oleate and TRAIL exhibited a significant increase of the fusion index (indicating the presence of multinucleated myotubes) as compared with those treated with oleate alone (Fig. 1H–I).

### TRAIL treatment promotes in vitro myogenic differentiation

**Fig. 2:**
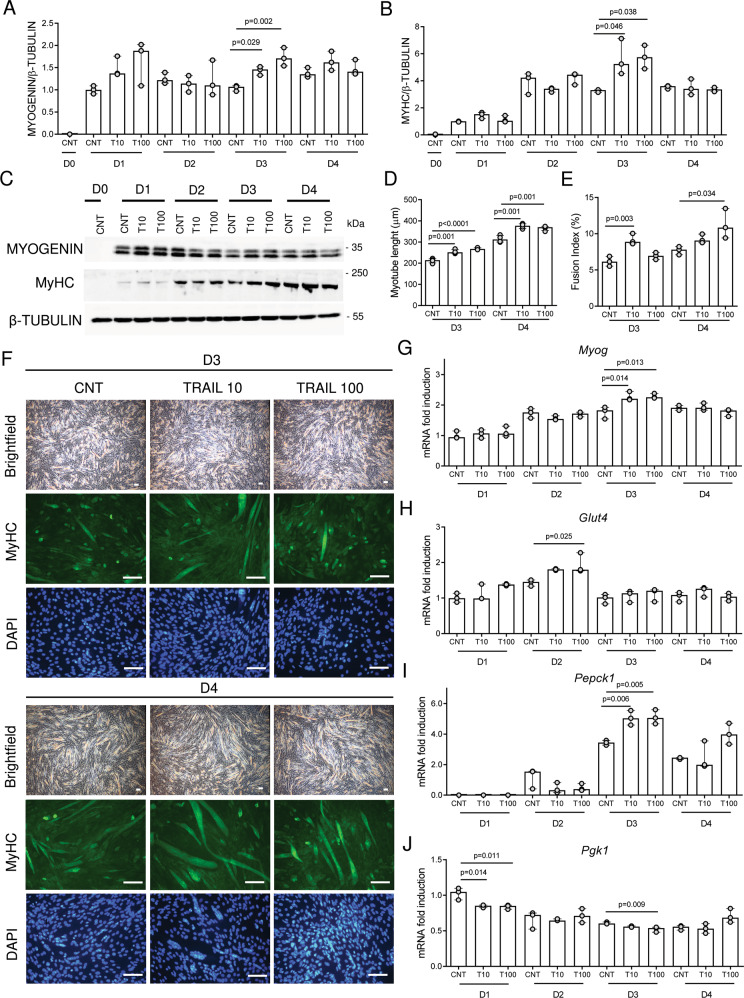
TRAIL promotes myogenic differentiation. Experiments were conducted in C2C12 cells during their differentiation. TRAIL treatment was renewed every 24 h between D0 and D4 (D is for day of differentiation). **A**–**B** Densitometric analysis and **C** representative blots of myogenin and MyHC protein expression. **D** Myotube length. **E** Fusion index. **F** Representative images of C2C12 cell morphology. Brightfield (upper panels), MyHC immunofluorescence (middle panels), DAPI staining (lower panels). Scale bar represents 100 µm. **G**–**J** Gene expression of *Myog*, *Glut4*, *Pepck1*, and *Pgk1*. Gene expression is reported as mRNA fold induction normalized to the mRNA level of CNT cells on D1. Results were obtained by 3–4 independent experiments and are presented as median with interquartile range. Significance was assessed with ANOVA and Tukey.

The following experiments were set up to evaluate whether TRAIL treatment could stimulate skeletal muscle differentiation. Daily exposure to TRAIL between D0–D4 (during myogenesis) significantly increased myogenin and MyHC protein expression, which are two transcription factors leading to myogenic differentiation (Fig. 2A–C). In particular, TRAIL stimulatory effect on myogenin and MyHC was seen on D1 and it became significant in all groups on D3. This was associated with an increase of myotube length and the fusion index on D3 and D4 (Fig. 2D–F). Consistent with these data, TRAIL significantly upregulated the gene expression of *Myog*, as well as the expression of *Glut4* and *Pepck1*, while it significantly downregulated *Pgk1* (Fig. 2G–J), indicating an effect not only on myogenic differentiation but also on glucose metabolism, which are two interconnected processes.

### *Dr5* silencing impairs in vitro myogenic differentiation

**Fig. 3:**
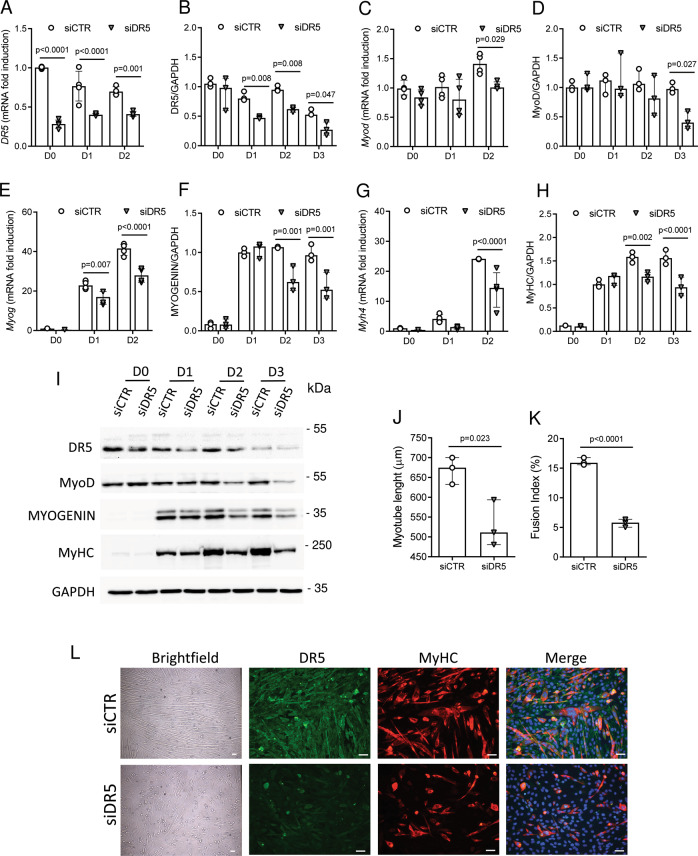
*Dr5* silencing significantly reduces myogenic differentiation. Experiments were conducted in C2C12 cells during their differentiation (D0–D3). *Dr5* was silenced before D0. **A**, **C**, **E**, **G** Gene expression of *Dr5*, *Myod*, *Myog, and Myh4*. Gene expression is reported as mRNA fold induction normalized to siCTR mRNA level on D0. **B**, **D**, **F**, **H** Quantification of DR5, MyoD, myogenin, and MyHC protein expression, and **I** representative blots. **J** Quantification of myotube length and **K** the fusion index on D3. Results were obtained by 3–4 independent experiments and are presented as median with interquartile range. Significance was assessed with ANOVA and Tukey tests (**A**–**H**), and **J**, **K** *t*-test. **L** Representative images of C2C12 cell morphology after *Dr5* silencing, as assessed on D3. Images are presented in brightfield, and/or after anti-DR5 staining (green staining), and/or after anti-MyHC staining (red staining). Scale bar = 50 µm.

DR5 is the only death receptor that mediates TRAIL signaling in mice [1, 2]. To determine if DR5 regulated skeletal muscle differentiation, C2C12 cells were transfected with siRNA to *Dr5*. Silencing efficiency was assessed by gene and protein expression, whereby we found that DR5 was significantly reduced (Fig. 3A, B). *Dr5* knockdown significantly reduced the gene and protein expression of MyoD (Fig. 3C, D), myogenin (Fig. 3E, F), and MyHC (Fig. 3G–I). Consistent with this, *Dr5* silencing was associated with a significant reduction of myotube length and the fusion index (Fig. 3J–L).

### TRAIL treatment promotes AKT phosphorylation and *Dr5* silencing reduces it

**Fig. 4:**
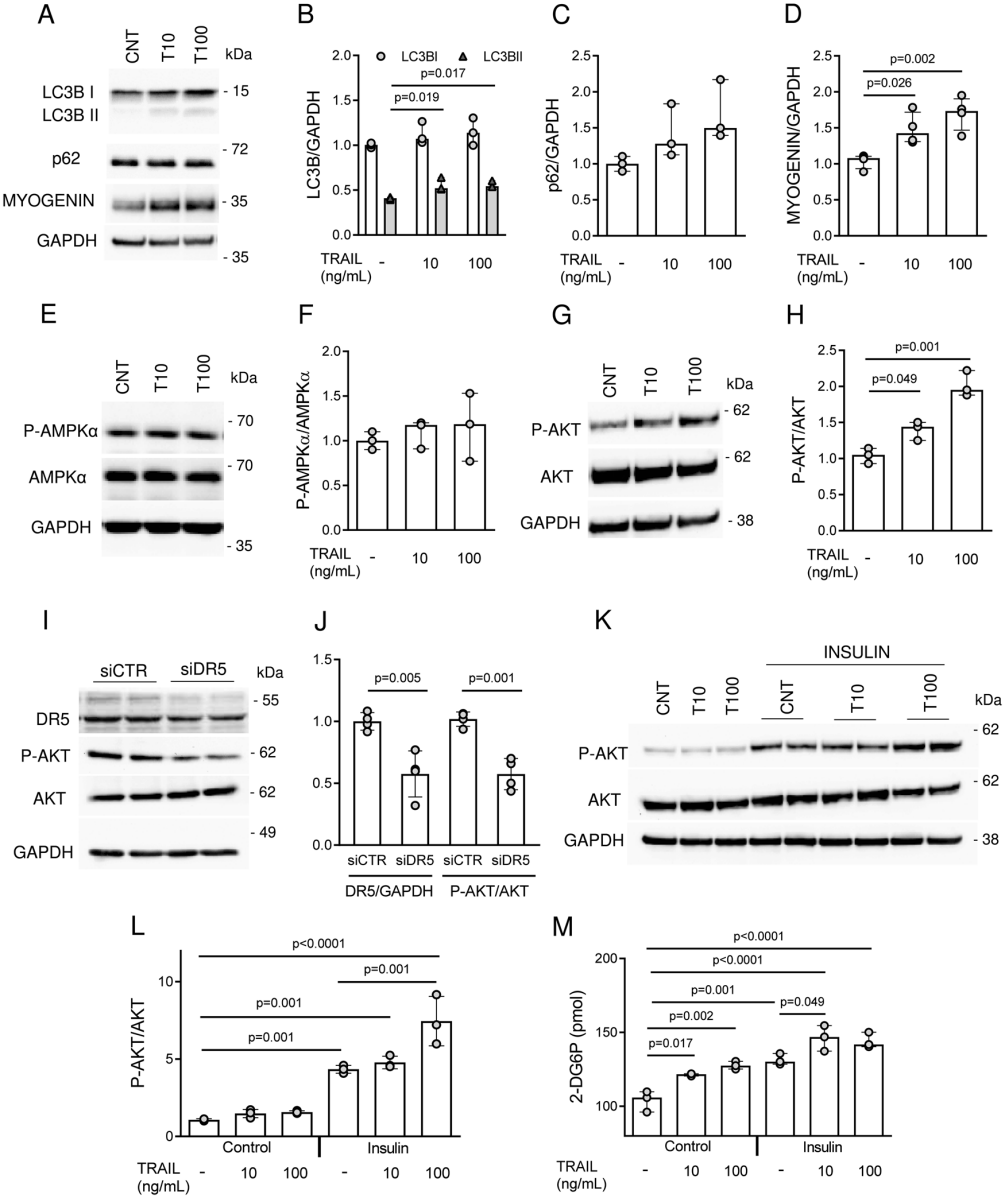
TRAIL/DR5 pathway regulates AKT phosphorilation. Experiments were conducted in C2C12 cells during their differentiation (D0–D3). TRAIL treatment was renewed every 24 h. **A** Representative blots and **B**–**D** quantification of LC3B-I and LC3B-II, p62, and myogenin expression on D3. **E**–**F** Representative blots and quantification of AMPKα and P-AMPKα expression on D3. **G**–**H** Representative blots and quantification of AKT and P-AKT expression on D3. **I**–**J** Representative blots and quantification of DR5, AKT, and P-AKT after *Dr5* silencing on D3. **K**–**L** Representative blots and quantification of AKT and P-AKT in C2C12 cells treated with TRAIL (24 h on D3) ± insulin (20 min on D5 after serum starvation). **M** Glucose uptake in C2C12 cells treated with TRAIL (24 h on D3) ± insulin (20 min on D5 after serum starvation). Results were obtained by 3–4 independent experiments and are presented as median with interquartile range. Significance was assessed with ANOVA followed by Tukey test, except for **J** where it was used the *t*-test.

In order to identify a possible mechanism underlying TRAIL effects on skeletal muscle differentiation, we tested if TRAIL contributed to a correct myogenesis through autophagy [20]. Cells were treated with TRAIL during their differentiation and harvested on D3. Our data showed that TRAIL treatment increased myogenin, but it did not increase autophagy. Although there was an increase of the amount of cleaved LC3B-II, which is a marker of autophagy, the expression of p62 did not significantly change upon TRAIL treatment, indicating that autophagy was not activated by TRAIL (Fig. 4A–D). Likewise, TRAIL effect on myogenesis did not seem to be mediated by the phosphorylation of AMPKα, which did not change upon TRAIL treatment [21] (Fig. 4E, F). By contrast, we found that TRAIL significantly increased AKT phosphorylation (Fig. 4G, H) and *Dr5* silencing significantly reduced it (Fig. 4I, J). Then, we evaluated the response to TRAIL treatment with the addition of insulin. Consistent with our previous data, we found that a 24-hour exposure to TRAIL on D3, followed by insulin treatment, significantly increased AKT phosphorylation (Fig. 4K, L). In the same experimental conditions, TRAIL significantly increased glucose uptake alone as well as combined with insulin (Fig. 4M).

### TRAIL treatment preserves myofiber size in HFD-fed mice and db/db mice

**Fig. 5:**
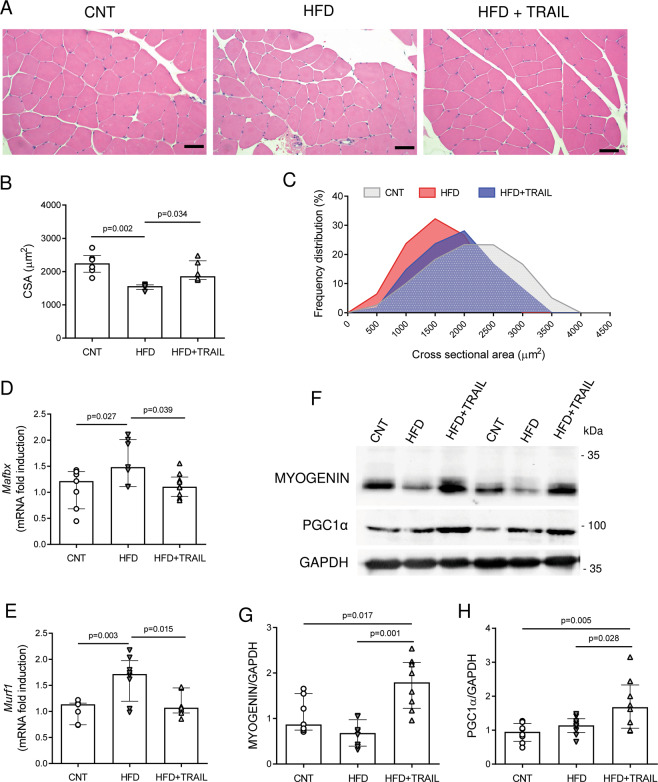
TRAIL treatment modulates myofiber size and function in HFD-fed mice. **A** Representative images and **B**–**C** quantification of quadricep myofiber cross-sectional area (CSA). Scale bar = 50 μm **D**–**E** Skeletal muscle gene expression of *Mafbx* and *Murf1*. Gene expression is reported as mRNA fold induction normalized to CNT. **F** Representative blots and **G**–**H** quantification of myogenin and PGC1α protein expression. CNT is for standard diet fed mice, HFD is for HFD-fed mice, and HFD + TRAIL is for HFD-fed mice treated with TRAIL. Results are presented as median with interquartile range. Significance was assessed with ANOVA and Tukey tests. *n* = 5–9 mice per group.

To evaluate whether TRAIL treatment had an effect on myofiber size in vivo, we measured the cross-sectional area of quadricep myofibers in two mouse models of T2DM, which is a condition associated with muscle mass loss [22]. In HFD-fed mice, TRAIL treatment significantly increased myofiber cross-sectional area as compared to untreated HFD-fed mice (Fig. 5A–C). In addition, TRAIL significantly reduced the expression of the atrophy-related genes *Mafbx* and *Murf1* (Fig. 5D, E), while it increased myogenin and PCG1α protein expression (Fig. 5F–H). In db/db mice, TRAIL treatment led to similar changes, as it significantly increased myofiber cross-sectional area, and it significantly reduced *Murf1* gene expression (Supplementary Fig. 1).

## Discussion

In this study, we explored TRAIL effects on skeletal muscle, finding that TRAIL treatment promoted myogenic differentiation. In vitro skeletal muscle differentiation is a multistep process that requires 2–5 days, where pluripotent mesodermal cells, called myoblasts, exit the cell cycle and differentiate into myocytes, which fuse to form multinucleated myotubes [23]. This process is coordinated by muscle regulatory factors, which are transcription factors that activate downstream genes to initiate muscle cell differentiation and include the myoblast determinantion protein (MyoD*)*, myogenin (MyoG), and myosin heavy chain (MyHC). *Myod* is the first factor that is expressed, which then induces *Myog*, another early target gene indicating the commitment of myoblastic cells to differentiation. Once myoblasts exit the cell cycle and differentiate, they upregulate the expression of other genes such as the *Myh* genes, which are required for muscle assembly and function. In parallel, differentiating myoblasts elongate and undergo fusion to form multinucleated myotubes. Our study shows for the first time that TRAIL treatment significantly upregulated myogenin and MyHC as well as other hallmarks of myogenic differentiation, such as myotube length and the fusion index.

TRAIL positive effect on skeletal muscle differentiation is consistent with its ability to stimulate not only pro-apoptotic but also pro-survival pathways [24, 25]. Previous works have shown that TRAIL stimulated the proliferation of several cell types, including endothelial, vascular smooth muscle cells, and fibroblasts [26–28]. We have also reported that TRAIL reduced the number of cardiac apoptotic cells in a model of diabetic cardiomyopathy [25]. Interestingly, Kavurma et al. observed that TRAIL effects change at different concentrations, as it induced vascular smooth muscle cell proliferation (and no apoptosis) at doses between 0.1 and 100 ng/mL, while it increased apoptosis and reduced their proliferation at concentrations greater than 400 ng/mL [29]. It has been argued that different concentrations of TRAIL might be accompanied with the redistribution of signaling molecules from non-rafts into lipid rafts, which determines if TRAIL has a pro-survival or pro-apoptotic final effect [30]. These observations might explain the findings by O’Flaherty et al., showing that TRAIL treatment induced an increase of the apoptosis associated with the earlier phases of skeletal muscle differentiation, given that in their study, myoblasts were cultured with TRAIL at greater concentrations (2000 ng/mL) than those that we used [31].

In addition, our study shows that during myogenic differentiation, TRAIL significantly changed the expression of some metabolic enzymes. We found an early and progressive decrease of *Pgk1* followed by a significant increase of *Glut4* and *Pepck*. Skeletal muscle differentiation and cellular metabolism are two interconnected processes, and our data are consistent with observations reported previously [32–34]. It has been shown that upon differentiation, the expression of *Glut4* as well as the expression of *Pepck* increase substantially [32, 33]. Both *Glut4* and *Pepck* have been implicated in the regulation of biosynthetic metabolic pathways in muscle growth [35]. By contrast, the formation of 3-phosphoglycerate, which is based on *Pgk1* activity, decreases significantly, such that the knockdown of *Pgk1* causes myoblast differentiation [34].

DR5 is the only death receptor that mediates TRAIL signaling in mice [1, 2]. Our observation that *Dr5* silencing significantly reduced the expression of muscle regulatory factors, as well as myotube length and the fusion index, indicates that what promotes skeletal muscle differentiation is the TRAIL/DR5 pathway. These data are in line with previous works showing that myoblasts expressing either a dominant negative mutant of DR5 or a dominant negative mutant of FADD, which is DR5 adapter protein, did not differentiate properly and exhibited decreased levels of MyoD [36].

In order to identify a possible mechanism underlying TRAIL effects on myogenic differentiation, we looked at a few pathways that might mediate this process. Fortini et al. demonstrated that autophagy is required to accomplish myotube fusion and that it is associated with AMPKα activation [20], which potentiates myogenin expression and myogenesis [37]. Our data show that TRAIL did not induce any further activation of autophagy [20] and/or AMPK [21], indicating that these are not the pathways mediating its effects. By contrast, TRAIL significantly increased AKT phosphorylation (while *Dr5* silencing reduced it), suggesting that it is AKT activation that mediates TRAIL/DR5 effect on skeletal muscle differentiation.

Phosphorilated AKT is crucially involved in skeletal muscle differentiation and growth. It mediates IGF-1 and insulin signaling, whereby it promotes protein synthesis, hypertrophy [38], and growth [39, 40], and it regulates glucose uptake and metabolism [41, 42]. The most abundant isoforms of AKT in skeletal muscle are AKT1 and AKT2. AKT1 deficiency impairs growth [39, 40], while AKT2 deficiency impairs glucose metabolism [41]. In this study, we report the ability of TRAIL (as well as DR5) to phosphorilate AKT during myogenic differentiation, which is consistent with what has been reported in other cell lines, such as endothelial cells [26] and Jurkat T cells [43]. In addition, here we found that TRAIL had a synergistic effect with insulin on insulin-stimulated AKT phosphorylation as well as glucose uptake. These findings might have significant implications for skeletal muscle homeostasis (including growth and metabolism) also in adult life.

Previous works have shown that TRAIL treatment significantly reduced insulin resistance in HFD-fed mice [12, 13], where it increased muscular ex vivo palmitate oxidation [12]. The synergistic effect of TRAIL with insulin on glucose uptake that we found in vitro can be one explanation. Another aspect can be the regulation of myofiber size, which is related to mitochondrial activity and insulin sensitivity [44]. In this study, we looked at myofiber size in vivo and found that HFD-fed mice as well as db/db mice had a significant reduction of myofiber size, and that TRAIL treatment significantly increased it in both models. This was associated with a reduction of the expression of the atrophy-related genes *Mafbx* and *Murf1*. In addition, in HFD-fed mice muscle cells, TRAIL significantly increased myogenin, which has been implicated in the regulation of myofier size, type, and homeostasis [17, 45], as well as PGC1α, which is a marker of mitochondrial biogenesis and oxidative metabolism [46]. These data show that TRAIL actions go beyond myofiber size and they extend to myofiber function.

In conclusion, our study demonstrates that TRAIL/DR5 pathway promotes AKT phosphorylation, skeletal muscle differentiation, and glucose uptake in vitro, as well as the maintenance of myofiber size in vivo. Our data shed light onto a pathway that might hold therapeutic potential not only for the metabolic disturbances but also for the muscle mass loss that are associated with diabetes or other pathological conditions.

## Supplementary information

Supplementary material

Supplementary Figure 1

## Data Availability

All data generated or analyzed during this study are included in this published article (and its supplementary information files).
