# Supplementary material for: TRAIL/DR5 pathway promotes AKT phosphorylation, skeletal muscle differentiation, and glucose uptake
Source: Cell Death Dis. 2021 Nov 16;12(12):1089. doi: 10.1038/s41419-021-04383-3 (PMC8599458; doi:10.1038/s41419-021-04383-3)
Supplement: Supplementary file 1 — Supplementary material [file 41419_2021_4383_MOESM1_ESM.docx]

**Supplementary Table 1. Primer list**

| **Target** | **Primer pair** |
| --- | --- |
| *Cpt1b* | (F) 5’-CCTCCGAAAAGCACCAAAAC -3’  (R) 5’-GCTCCAGGGTTCAGAAAGTAC -3’ |
| *Glut4* | (F) 5'-TGTCGCTGGTTTCTCCAACTG-3'  (R) 5'-CCATACGATCCGCAACATACTG-3' |
| *Pepck1* | (F) 5’-CCATCCCAACTCGAGATTCTG-3’  (R) 5’- CTGAGGGCTTCATAGACAAGG-3’ |
| *Pgc1α* | (F) 5’-TGATGTGAATGACTTGGATACAGACA-3’  (R) 5’- GCTCATTGTTGTACTGGTTGGATATG-3’ |
| *Pgk1* | (F) 5’-AACCTCCGCTTTCATGTAGAG-3’  (R) 5’-GACATCTCCTAGTTTGGACAGTG-3’ |
| *Pparg* | (F) 5'-TGTCGGTTTCAGAAGTGCCTTG-3'  (R) 5'-TTCAGCTGGTCGATATCACTGGAG-3' |
| *MyoD* | (F) 5’-AACTGTCCTTTCGAAGCCGT -3’  (R) 5’-TTGGGGCTGGATCTAGGACA -3’ |
| *Myog* | (F) 5’-CTGCCTAAAGTGGAGATCCTG -3’  (R) 5’-TGGGAGTTGCATTCACTGG -3’ |
| *Myh4* | (F) 5'-CAAAAGCAAAGGGAAGAGCAG -3'  (R) 5'-AGCAGAGTTCAGACTTGTCAG -3' |
| *Mafbx* | (F) 5'-AGAACAGCAAAACCAAAACTCAG-3'  (R) 5'-GTCGAGAAGTCCAGTCTGTTG-3' |
| *Murf1* | (F) 5'-GCTACCTTCCTCTCAAGTGC-3'  (R) 5'-CCTCTGCTATGTGTTCTAAGTCC-3' |
| *Gapdh* | (F) 5’-AAATGGTGAAGGTCGGTGTG-3’  (R) 5’-TGAAGGGGTCGTTGATGG-3’ |
| *Rpl27* | (F) 5'-TCATGCCCACAAGGTACTCTGT-3'  (R) 5'-CTGGCCTTGCGCTTCAAA-3' |

Supplementary Table 2: Primary antibody list

| **Antibody** | **Host** | **Dilution** | **Provider** | **Category n°** |
| --- | --- | --- | --- | --- |
| Myosin Heavy Chain | Mouse | 1:1000 | Merck  (Darmstadt, Germany) | 05-716 |
| MyoD | Mouse | 1:100 | Santa Cruz Biotechnology (Dallas, TX, USA) | sc-377460 |
| Myogenin | Mouse | 1:200 | Santa Cruz Biotechnology | sc-52903 |
| DR5 | Rabbit | 1:500 | abcam (Cambridge, United Kingdom) | ab8416 |
| Phospho-Akt (Ser473) | Rabbit | 1:1000 | Cell Signaling Technology (Danvers, MA, USA) | 9271 |
| Akt | Rabbit | 1:1000 | Cell Signaling Technology | 9272 |
| Phospho-Ampkα (Thr172) | Rabbit | 1:1000 | Cell Signaling Technology | 2535 |
| Ampkα | Rabbit | 1:1000 | Cell Signaling Technology | 2532 |
| LC3B | Rabbit | 1:1000 | GeneTex  (Irvine, CA, USA) | GTX127375 |
| p62-SQSTM1 | Rabbit | 1:1000 | Merck | P0067 |
| PGC1 alpha | Rabbit | 1:1000 | Thermo Fisher Scientific  (Waltham, MA, USA) | PA5-38021 |
| GAPDH | Rabbit | 1:5000 | Cell Signaling Technology | 2118 |
| β-Tubulin | Rabbit | 1:1000 | Cell Signaling Technology | 2128 |

**Supplementary Figure 1. TRAIL treatment prevents muscle atrophy in db/db mice.** (**A**) Representative images and (**B-C**) quantification of quadricep myofiber cross-sectional area. (**D-E**) Skeletal muscle gene expression of *Mafbx* and *Murf1*. Gene expression is reported as mRNA fold induction normalized to db/H. Results are presented as median with interquartile range. Significance was assessed with ANOVA and Tukey tests (B and D), and Kruskall-Wallis and Dunn’s tests (E). n=4-6 mice per group.
