# Supplementary figures and images for: TRAIL/DR5 pathway promotes AKT phosphorylation, skeletal muscle differentiation, and glucose uptake
Source: Cell Death Dis. 2021 Nov 16;12(12):1089. doi: 10.1038/s41419-021-04383-3 (PMC8599458; doi:10.1038/s41419-021-04383-3)

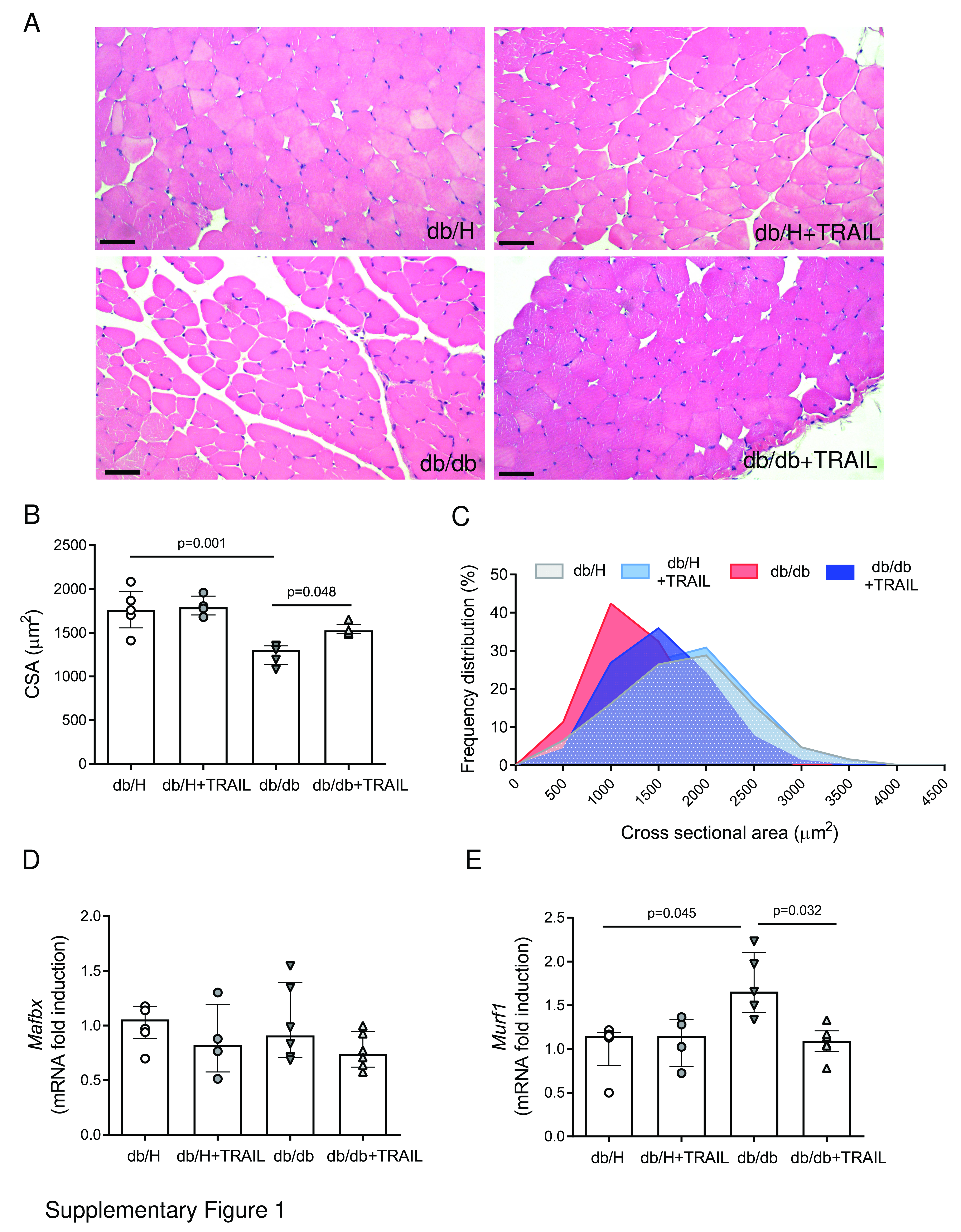

Supplement: Supplementary file 2 — Supplementary Figure 1 [file 41419_2021_4383_MOESM2_ESM.tif]
